# Supplementary material for: Service user perceptions of smoking cessation in residential substance use treatment
Source: PLoS One. 2022 Jun 22;17(6):e0270045. doi: 10.1371/journal.pone.0270045 (PMC9216544; doi:10.1371/journal.pone.0270045)
Supplement: Attachment — Submitted filename: Service user perceptions of smoking cessation in residential substance use treatment- review.docx [file pone.0270045.s001.docx]

**Service user perceptions of smoking cessation in residential substance use treatment**

**Reviewer:** Dr Lisa Dannatt; University of Cape Town, Faculty of Health Sciences; Department of Psychiatry and Mental Health

**General comments:**

Thank you for asking me to review this manuscript. It is well thought out and well written. Please carefully consider by suggestions below.

I do not have any conflicts of interest in reviewing this manuscript.

**Abstract:**

The abstract is clear and concise with detail regarding background, methods, results, and conclusion.

**Introduction:**

The introduction gives a clear indication of what is known and not known. I would suggest the following edits

Line 66: Tobacco kills an estimated eight million people per year, worldwide. Suggest: Tobacco use carries a significant mortality of an estimated eight million people per year, worldwide.

Line 67-73: Focus is on smoking among alcohol users. What about other substances? Or was the target population only alcohol users undergoing treatment? If so, please clarify in the abstract.

Line 86: Referencing differs from the rest of the manuscript

Line 97: Despite evidence to support cessation in substance use treatment, little research is available. This sentence seems contradictory. Can it be clarified? A strong introductory sentence will lend weight to this important paragraph.

**Methods**

Methods are clear and concise. Please clarify the comments below.

Line 114: Ethical approval for this study was obtained from a university research ethics committee. Should you indicate which university? What is expected by the journal?

I also wondered if you could clarify what residential treatment looks like e.g.- 6-week elective admission to a CBT based program.

**Results:**

**Profile-** would a table be helpful here? Again, what are the expectations of the journal?

Results are well structured into coherent themes with lovely supporting quotes from participants. It gives a clear sense of the collected qualitative data.

Would it be useful to use table above and number participants e.g. Participant 1 would link to table as 60 year old smoker on treatment?

Line 266- numbered reference?

**Discussion:**

Back up your statements with existing literature or highlight what is new.

Line 300-304: Suggest highlighting these contradictory viewpoints by citing existing literature as mentioned in the intro.

Line 345- 350: Any existing literature? Is this novel?

Line 365-367: also see existing literature comments

I would also add specific headings for conclusion and recommendations (for both services and future research and be more explicit about limitations
