# Supplementary material for: Service user perceptions of smoking cessation in residential substance use treatment
Source: PLoS One. 2022 Jun 22;17(6):e0270045. doi: 10.1371/journal.pone.0270045 (PMC9216544; doi:10.1371/journal.pone.0270045)
Supplement: Attachment — Submitted filename: Response to reviewers .docx [file pone.0270045.s002.docx]

**Response to Reviewer’s Comments**

We would like to thank both the reviewers for their comprehensive and constructive feedback. As directed we have used track changes in our resubmission and below we detail how we have responded to the points raised. We hope you find these amendments satisfactory.

**Reviewer 1 Comments**

**Introduction:**

Line 66: Tobacco kills an estimated eight million people per year, worldwide. Suggest: Tobacco use carries a significant mortality of an estimated eight million people per year, worldwide.

Thank you for the suggestion, this line has been amended accordingly.

Line 67-73: Focus is on smoking among alcohol users. What about other substances? Or was the target population only alcohol users undergoing treatment? If so, please clarify in the abstract.

The study included adults in treatment for any substance, this section has been updated to include drug and alcohol users as suggested, and the abstract amended accordingly.

Line 86: Referencing differs from the rest of the manuscript

This has been corrected.

Line 97: Despite evidence to support cessation in substance use treatment, little research is available. This sentence seems contradictory. Can it be clarified? A strong introductory sentence will lend weight to this important paragraph.

This sentence has been adjusted to specify the lack of research on service user perspectives on this issue.

**Methods**

Line 114: Ethical approval for this study was obtained from a university research ethics committee. Should you indicate which university? What is expected by the journal?

Ethical approval was obtained from Liverpool John Moores Research Ethics Committee, reference number: 17/PBH/005.

I also wondered if you could clarify what residential treatment looks like e.g.- 6-week elective admission to a CBT based program.

Thank you for this suggestion, a line and a reference have been added to clarify this: The treatment services included were based on a therapeutic community model (Campling, 2001), and involved a voluntary 3–9-month admission to a group-based program.

**Results:**

**Profile-** would a table be helpful here? Again, what are the expectations of the journal?

Given the small sample size, it was felt that the information in the main text was sufficient and a table here would be repetitive. This does not appear to be an expectation of the journal, and as such has not been added here.

Would it be useful to use table above and number participants e.g., Participant 1 would link to table as 60-year-old smoker on treatment?

Thank you for this suggestion, but as above, given the small sample size it was felt that this might be too identifying for participants and as such has not been included. Previous qualitative work published by the journal has used a similar format.

Line 266- numbered reference?

This has been corrected.

**Discussion:**

Back up your statements with existing literature or highlight what is new.

Line 300-304: Suggest highlighting these contradictory viewpoints by citing existing literature as mentioned in the intro.

This has been amended to reflect the existing literature as suggested.

Line 345- 350: Any existing literature? Is this novel?

As suggested by reviewer 2, the line about increased harms of rolling tobacco has been removed due to insufficient evidence on these. Regarding the cost being a motivating factor, I have mentioned that this is established in literature, and provided a reference which demonstrates that cost of tobacco is a motivating factor in both quitting smoking and switching to e-cigarette use.

Line 365-367: also see existing literature comments

Another line and reference have been added here: This is also supported by existing literature, which highlights the importance of psychological support for quitting smoking.

I would also add specific headings for conclusion and recommendations (for both services and future research and be more explicit about limitations

Thank you for these suggestions, headings for conclusion and recommendations have been added, as have elaborations on the limitations of the study, which include the small sample size and length of time since treatment completion.

**Reviewer 2 Comments**

Reviewer #2: 1. Line 44: the word "centers" missing

The word ‘services’ has been added here for clarity.

2. There were a couple of references that were written in this pattern; Reid et al (Reid et al., 2008). This should be corrected throughout the manuscript

Thank you for highlighting this, it has been corrected.

3. Lines 127-128: "Saturation was considered to be reached as no new codes were identified in the final transcripts analysed." I do not think saturation is achieved in analysis but interviews. I do not think this is the right use of the concept of 'saturation' in qualitative research.

Saturation in qualitative research can have different meanings, the stance appropriated here is defined by Urquhart (2013) as “the point in coding when you find that no new codes occur in the data. There are mounting instances of the same codes, but no new ones”.

4. Line 129: add the word "by" after "checked" in the sentence "were checked a member..."

This has been added.

5. Lines 134-140: please state why the participants were in rehabilitation? For Alcohol only, or other drugs or both?

Participants were admitted to the treatment services for any substance, although all the participants were in treatment for alcohol (including three for both alcohol and drug use). This has been added here with numbers:

“Seven were in treatment for alcohol use only, the remaining three used both alcohol and drugs.”

6. Line 154: in a few places in the manuscript, no specific number of participants were mentioned. Since the number are so few, it would be good to see the number of participants replacing words such as "several" or "some".

This is a useful suggestion, and has been addressed in lines 156, 157, 169, 182, 237, 265 and 271 of the updated manuscript; hopefully this adds weight to the participant perspectives.

7. Lines 143ff: Many of the headings do not seem to work well with the content in the results section. Consider reviewing the themes to more appropriately describe the content. For example, "smoking is the norm in treatment centers" could be rephrased as "Smoking seen as a norm in treatment centers" Or "The culture of smoking in treatment centers"

These headings have been reconsidered and two updated accordingly, to ensure that the headings match the content.

‘Smoking is the norm in treatment’ has been changed to ‘the culture of smoking in treatment services’.

‘Smoking as different to other drugs’ had been updated to ‘Relationship between tobacco and other substances’.

8 Line 270: the methods section says there were only service users in the sample but this line contradicts that.

Line 143 of the updated document explains that five participants had previously been in residential treatment services but were currently working or volunteering in services, I have removed this from line 270 as it is not needed. It is mentioned again in line 208, so I have amended this too.

9. Please summarize the findings at the end of the results section

A brief summary of the findings has been added at the end of this section as suggested.

10. Lines 311 -316: the findings do not speak to harm reduction. Also, there is not enough evidence to make a claim that the user of e-cig in this sample did that to reduce harm. A change of addictive substance is not harm reduction.

This section has been amended to suggest that the popularity of e-cigarettes could be utilised as a cessation tool, as suggested by NICE guidelines in the UK.

11. Lines 312-313: sentence beginning in 312; I didn't see this in the results. Only saw a change of product to save money.

This sentence has been changed as in the above comment, to remove the mention of harm reduction as the results did not explore motivations to switch from smoking to vaping in enough detail to assume it was to reduce harm. Regardless of their reasoning, some participants had changed from smoking cigarettes to using e-cigarettes, and this could be utilised to encourage the use of e-cigarettes as a smoking cessation tool. This approach is recommended in the UK by the NHS and Public Health England.

12. Lines 313-316: "Although they remain controversial as a cessation tool (Hartmann-Boyce et al., 2021), e-cigarettes allow smokers to retain their personal and social identity as a smoker, whilst reducing the harms associated with tobacco
consumption." The last phrase of this statement is not factual. There are several studies showing the harms caused by e-cigarettes. Though studies are on-going, there is presently sufficient evidence to confirm that e-cigarettes are harmful to health in many ways like traditional cigarettes.

This line has been amended to acknowledge that whilst neither tobacco or e-cigarettes are safe and good for health, current available evidence suggests that vaping may be less harmful than tobacco smoking and therefore could be a better alternative. This stance is backed by the Centres for Disease Control and Prevention in the USA as well as the NHS and PHE in the UK. We appreciate reviewer 2’s stance on this issue but feel that our explanation as supported by evidence us sufficient.

13. Lines 340-343: I'm not sure this reason explains fully the issue presented here. I think awareness of the harms caused by tobacco and how soon it can affect them may be responsible. The greater risk of death in the long term may be responsible as opposed to visible immediate consequences of alcohol abuse. Cheeta et al 2018 as quoted in the next sentence explains it better.

Due to the focus of treatment on alcohol / drugs, there was an increased awareness of both the short- and long-term damages done by these substances, whereas there appeared to be a more vague and distant perception of the risks of tobacco smoking. Although all the participants knew smoking was bad for their health, it was generally seen as something that would be dealt with later on, if at all.

14. Lines 347-348: Please reference this statement or delete it. There is no evidence that rolling tobacco and illicit tobacco are more dangerous to health.

This statement has been deleted as suggested.

15. Add sections on limitations and conclusion

Thank you for this helpful suggestion, these sections have been added and hopefully add clarity to the manuscript.

Urquhart, C. Grounded Theory for Qualitative Research: A Practical Guide. Sage, Thousand Oaks (2013)
